# Association and Host Selectivity in Multi-Host Pathogens

**DOI:** 10.1371/journal.pone.0000041

**Published:** 2006-12-20

**Authors:** José M. Malpica, Soledad Sacristán, Aurora Fraile, Fernando García-Arenal

**Affiliations:** 1 Departamento de Biotecnología, Instituto Nacional de Investigación Agraria y Alimentaria Madrid, Spain; 2 Departamento de Biotecnología y Centro de Biotecnología y Genómica de Plantas, Universidad Politécnica de Madrid Madrid, Spain; Oxford University, United Kingdom

## Abstract

The distribution of multi-host pathogens over their host range conditions their population dynamics and structure. Also, host co-infection by different pathogens may have important consequences for the evolution of hosts and pathogens, and host-pathogen co-evolution. Hence it is of interest to know if the distribution of pathogens over their host range is random, or if there are associations between hosts and pathogens, or between pathogens sharing a host. To analyse these issues we propose indices for the observed patterns of host infection by pathogens, and for the observed patterns of co-infection, and tests to analyse if these patterns conform to randomness or reflect associations. Applying these tests to the prevalence of five plant viruses on 21 wild plant species evidenced host-virus associations: most hosts and viruses were selective for viruses and hosts, respectively. Interestingly, the more host-selective viruses were the more prevalent ones, suggesting that host specialisation is a successful strategy for multi-host pathogens. Analyses also showed that viruses tended to associate positively in co-infected hosts. The developed indices and tests provide the tools to analyse how strong and common are these associations among different groups of pathogens, which will help to understand and model the population biology of multi-host pathogens.

## Introduction

Pathogens have highly variable host ranges: in natural conditions some infect only one or a few related species (i.e., specialist pathogens) while other can infect a wide range of hosts belonging to different taxonomic groups (i.e., multi-host or generalist pathogens). A large fraction of described pathogens of humans, animals and plants are generalists [1]–[3]. The ability to infect different hosts conditions the epidemiology and pathogenicity of generalist pathogens and, therefore, is highly relevant for pathogen management and disease control [1], [4]. The distribution of multi-host pathogens over their host range, i.e. the frequency of infection in the various host species within an ecosystem, may vary largely, which could determine the population dynamics and structure of the pathogen. The distribution of a pathogen species over its host range may also determine important aspects of its biology in hosts significant from an anthropocentric viewpoint (i.e. target hosts), such as reservoirs and inoculum sources, emergence and re-emergence, population thresholds for disease invasion or critical community size for disease persistence [e.g., [1], [4]–[7]].

Animal or plant species may be hosts for a range of pathogens, and most host populations encounter a large number of different pathogen species [8]. For significant host species, there is abundant evidence of differences in the infection frequency of the various pathogen species present in an ecosystem. The distribution of pathogens over their hosts, and the distribution of different pathogens within a host species, will affect the frequency of multiple infection of an individual host by different pathogens. Multiple infection may have important consequences for the infected hosts, for the pathogens, and for host-pathogen co-evolution [8], [9]. In the host, frequent co-infections may lead to heterozygote superiority against multiple pathogens and contribute to the persistence in host populations of alleles conferring susceptibility to disease [10]. In multiple infected hosts, pathogens can cooperate or can compete for host resources, which will affect each other's fitness. Hence, multiple infections will be a factor in pathogen evolution. Theoretical analyses predict that the within-host dynamics of microparasites in multiple infected hosts may have important consequences in the evolution of their virulence [11]–[14], and there is evidence that multiple infection may result in either increased or reduced virulence [e.g., [15]–[17]]. Multiple infection of a host may also directly affect the genetic diversity of the pathogen population, as co-infection is a prerequisite for genetic exchange between different pathogen species or strains. Also, infection by one pathogen may result in an increased host susceptibility to a second pathogen, a common phenomenon named facilitation or predisposition by animal and plant pathologists, respectively [8], [18].

In spite of its potential impact on pathogenicity, evolution, epidemiology and control, the distribution of pathogens over their host range and the occurrence of co-infections have been largely overlooked, and most research on pathogen ecology and epidemiology has dealt with specific pathogen-host interactions [8]. To our knowledge, it has not been analysed whether the distribution of pathogens over their host range is random or, alternatively, associations between pathogens and hosts occur, neither has been addressed whether host co-infection by different pathogens is random or associations between pathogens occur in particular hosts. Here we address these issues.

First, we propose indices for the observed patterns of host infection by different pathogens, and the observed patterns of co-infection, and tests to analyse if they conform to the null hypothesis of randomness or reflect associations. Second, we apply these tests to data on the prevalence of five insect-borne virus species in wild plant species within an agroecosystem in Central Spain. Results of these analyses uncover patterns that, if general, would be highly relevant to understand the ecology and evolution of pathogens.

## Results

### Association between viruses and hosts, and among viruses, in weeds in Central Spain

Between January 2000 and December 2002, 2275 samples from 56 weed species or genera pertaining to 21 dicotyledonous plant families, were collected and analyzed for infection by the aphid transmitted viruses *Alfalfa mosaic virus* (AMV, genus *Alfamovirus*, family *Bromoviridae*), *Beet western yellows virus* (BWYV, genus *Polerovirus*, family *Luteoviridae*), *Cucumber mosaic virus* (CMV, genus *Cucumovirus*, family *Bromoviridae*), and *Watermelon mosaic virus* (WMV, genus *Potyvirus*, family *Potyviridae*), and by the thrips-transmitted *Tomato spotted wilt virus* (TSWV, genus *Tospovirus*, family *Bunyaviridae*) [19]. Except for TSWV, which has a single-stranded RNA genome of negative and ambisense polarity, all other viruses have single-stranded RNA genomes of messenger polarity. AMV, CMV and WMV are transmitted by aphids in a non-persistent manner, i.e. the virus is retained in the distal structures of the aphid mouth parts for short period of time. BWYV is transmitted in a circulative, non-propagative manner, i.e., the virus penetrates through the gut wall into the haemocoel of the insect vector, and circulates with the haemolymph to reach the salivary glands, from where it is inoculated into new plants. TSWV follows a similar path within the thrips body, but infects and multiplies in the insect cells [19]. All five viruses cause important diseases in vegetable crops world-wide, including the studied region in Central Spain, but infection in the analysed wild hosts was asymptomatic. Table 1 shows the number of samples analysed and the number of infected plants by each of these five virus species, in single or multiple infection, in the 21 most frequently found plant species in three monitored habitats (see Methods) for the analysed period.

**Table 1:**
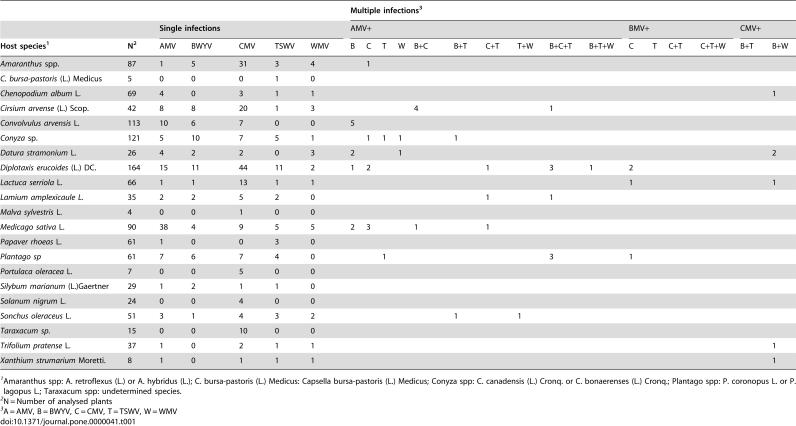
Number of single and multiple infections with AMV, BWYV, CMV, TSWV and WMV in twenty one weed species

							Multiple infections^3^															
		Single infections					**AMV+**										**BMV+**				**CMV+**	
Host species^1^	N^2^	**AMV**	**BWYV**	**CMV**	**TSWV**	**WMV**	**B**	**C**	**T**	**W**	**B+C**	**B+T**	**C+T**	**T+W**	**B+C+T**	**B+T+W**	**C**	**T**	**C+T**	**C+T+W**	**B+T**	**B+W**
*Amaranthus* spp.	87	1	5	31	3	4		1									3					3
*C. bursa-pastoris* (L.) Medicus	5	0	0	0	1	0																
*Chenopodium album* L.	69	4	0	3	1	1																1
*Cirsium arvense* (L.) Scop.	42	8	8	20	1	3					4				1		3					
*Convolvulus arvensis* L.	113	10	6	7	0	0	5															
*Conyza* sp.	121	5	10	7	5	1		1	1	1		1						2				
*Datura stramonium* L.	26	4	2	2	0	3	2			1												2
*Diplotaxis erucoides* (L.) DC.	164	15	11	44	11	2	1	2					1		3	1	2			1	3	
*Lactuca serriola* L.	66	1	1	13	1	1											1					1
*Lamium amplexicaule L.*	35	2	2	5	2	0							1		1							
*Malva sylvestris* L.	4	0	0	1	0	0																
*Medicago sativa* L.	90	38	4	9	5	5	2	3			1		1						1		1	2
*Papaver rhoeas* L.	61	1	0	0	3	0																
*Plantago sp*	61	7	6	7	4	0			1						3		1					
*Portulaca oleracea* L.	7	0	0	5	0	0																
*Silybum marianum* (L.)Gaertner	29	1	2	1	1	0																
*Solanum nigrum* L.	24	0	0	4	0	0																
*Sonchus oleraceus* L.	51	3	1	4	3	2						1		1							1	
*Taraxacum sp.*	15	0	0	10	0	0																
*Trifolium pratense* L.	37	1	0	2	1	1																1
*Xanthium strumarium* Moretti.	8	1	0	1	1	1																1

1 Amaranthus spp: A. retroflexus (L.) or A. hybridus (L.); C. bursa-pastoris (L.) Medicus: Capsella bursa-pastoris (L.) Medicus; Conyza spp: C. canadensis (L.) Cronq. or C. bonaerenses (L.) Cronq.; Plantago spp: P. coronopus L. or P. lagopus L.; Taraxacum spp: undetermined species.

2 N = Number of analysed plants

3 A = AMV, B = BWYV, C = CMV, T = TSWV, W = WMV

**Figure 1:**
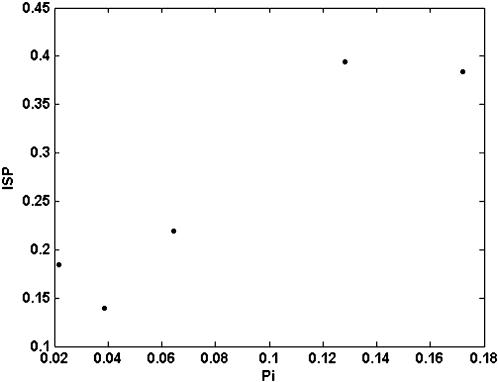
Relationship between average prevalence (P_i_) and the index of selectivity of the pathogen (ISP) for five virus species.

**Figure 2:**
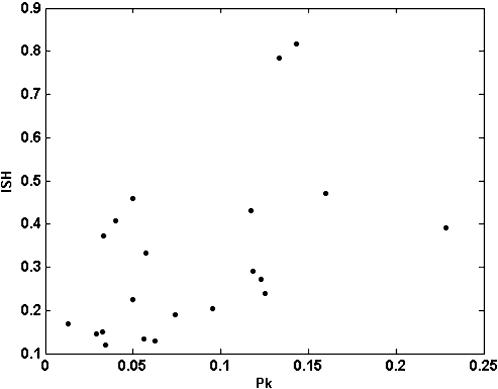
Relationship between average prevalence (P_k_) of virus infection and the index of selectivity of the host (ISH) for twenty one weed species.

To this data set tests for association between hosts and pathogens (see Methods) were applied. The index of selectivity of pathogen (ISP), and its significance, is shown in Table 2 for the five viruses. The distribution of three of five analysed viruses over their hosts was significantly non-random, i.e. some of the available hosts were preferentially infected. Fig. 1 shows the relationship between prevalence and the ISP for the five viruses. A positive correlation was found for both parameters (r = 0.9347, P = 0.0189 in a Spearman rank correlation test), i.e., the more host-selective viruses were those with a highest prevalence in the analysed ecosystem. Similarly, the index of selectivity of the host (ISH), and its significance, was calculated for the 21 host plant species in Table 1, and values are shown in Table 3. For about half (9/21) of the analysed hosts (*Amaranthus* spp., *Cirsium arvense, Convolvulus arvensis, Diplotaxis erucoides, Lactuca serriola, Medicago sativa, Portulaca oleracea, Solanum nigrum* and *Taraxacum* spp.) differences in the prevalence of the five viruses departed significantly from random. Fig. 2 shows the relationship between virus prevalence and the ISH for the 21 host species. Again, a positive correlation between both parameters was found (r = 0.5161, P = 0.0166, in a Spearman rank correlation test), i.e., the more virus-selective hosts were those with a higher prevalence of virus infection. The relationships between prevalence and selectivity for viruses and hosts were not due to a coincidence in the frequency of infection among hosts by different viruses, as shown by a contingency analysis of counts of infected hosts by the different viruses (P<10^−4^).

**Table 2:**
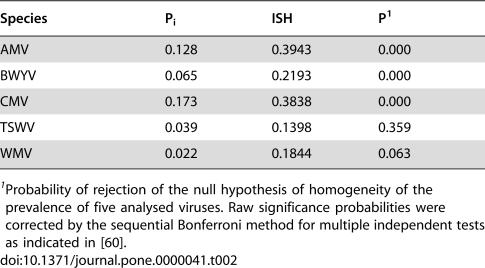
Average prevalence (P_i_), and index of selectivity of the pathogen (ISP) for five virus species.

Species	P_i_	ISH	P^1^
AMV	0.128	0.3943	0.000
BWYV	0.065	0.2193	0.000
CMV	0.173	0.3838	0.000
TSWV	0.039	0.1398	0.359
WMV	0.022	0.1844	0.063

1 Probability of rejection of the null hypothesis of homogeneity of the prevalence of five analysed viruses. Raw significance probabilities were corrected by the sequential Bonferroni method for multiple independent tests as indicated in [60].

**Table 3:**
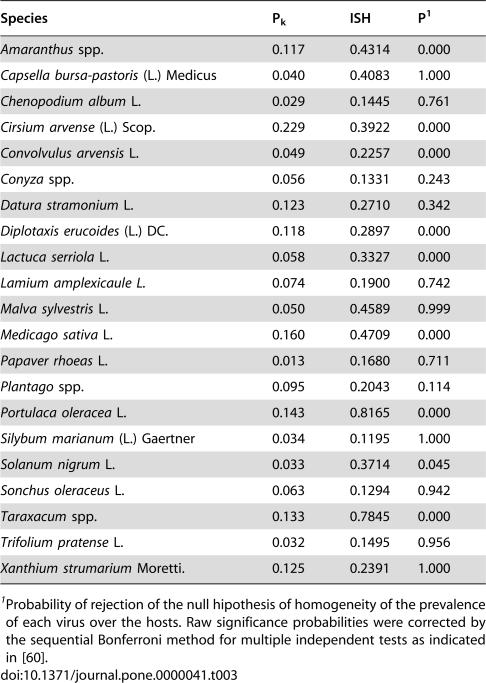
Average prevalence of virus infection (P_k_), and index of selectivity of the host (ISH) for twenty one weed species.

Species	P_k_	ISH	P^1^
*Amaranthus* spp.	0.117	0.4314	0.000
*Capsella bursa-pastoris* (L.) Medicus	0.040	0.4083	1.000
*Chenopodium album* L.	0.029	0.1445	0.761
*Cirsium arvense* (L.) Scop.	0.229	0.3922	0.000
*Convolvulus arvensis* L.	0.049	0.2257	0.000
*Conyza* spp.	0.056	0.1331	0.243
*Datura stramonium* L.	0.123	0.2710	0.342
*Diplotaxis erucoides* (L.) DC.	0.118	0.2897	0.000
*Lactuca serriola* L.	0.058	0.3327	0.000
*Lamium amplexicaule L.*	0.074	0.1900	0.742
*Malva sylvestris* L.	0.050	0.4589	0.999
*Medicago sativa* L.	0.160	0.4709	0.000
*Papaver rhoeas* L.	0.013	0.1680	0.711
*Plantago* spp.	0.095	0.2043	0.114
*Portulaca oleracea* L.	0.143	0.8165	0.000
*Silybum marianum* (L.) Gaertner	0.034	0.1195	1.000
*Solanum nigrum* L.	0.033	0.3714	0.045
*Sonchus oleraceus* L.	0.063	0.1294	0.942
*Taraxacum* spp.	0.133	0.7845	0.000
*Trifolium pratense* L.	0.032	0.1495	0.956
*Xanthium strumarium* Moretti.	0.125	0.2391	1.000

1 Probability of rejection of the null hipothesis of homogeneity of the prevalence of each virus over the hosts. Raw significance probabilities were corrected by the sequential Bonferroni method for multiple independent tests as indicated in [60].

**Figure 3:**
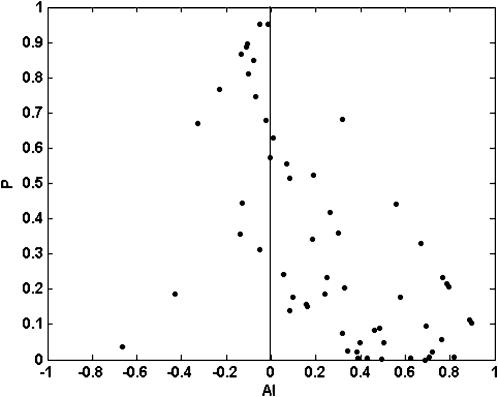
Distribution of the values of the association index (AI) and their individually associated probabilities of significance, for 68 virus-host plant systems.

For 16 of the 21 plant species in Table 1, co-infection with more than one of the five viruses occurred. For these 16 plant species, 102 plants were infected by at least one virus out of 1060 analysed plants (Table 4). The above described test of association between pathogens was applied to this set. The data in Table 4 showed a tendency of the analysed viruses to associate positively: the distribution of the association index (AI) was skewed towards positive values (Fig. 3) so that out of 68 AIs computed for the five viruses in 16 plant species, 47/68 (more than two thirds) were positive and 21/68 were negative. Moreover, there was a conspicuous tendency of the positive AI values to have smaller probabilities (r = −0.6575, P<10^−4^, in a Spearman rank correlation test). When the pooled sample from the sixteen plant species was considered, the AI was positive and significantly different from zero for each of the five viruses, i.e. each of the five viruses was found in co-infection with a frequency significantly higher than expected from the null hypothesis of independence of infection. However, this was not so when the data for each of the sixteen plant species were analyzed separately. Hence the association analysis uncovered two patterns that were not obvious: i) a general tendency of the analysed viruses to associate positively, ii) association depended on both the plant and the virus species.

**Table 4:**
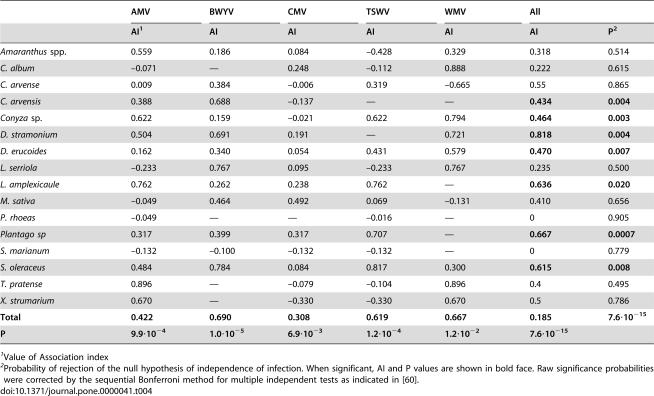
Analysis of association among five virus species in sixteen host plant species

	AMV	BWYV	CMV	TSWV	WMV	All	
	AI^1^	AI	AI	AI	AI	AI	P^2^
*Amaranthus* spp.	0.559	0.186	0.084	–0.428	0.329	0.318	0.514
*C. album*	–0.071	—	0.248	–0.112	0.888	0.222	0.615
*C. arvense*	0.009	0.384	–0.006	0.319	–0.665	0.55	0.865
*C. arvensis*	0.388	0.688	–0.137	—	—	**0.434**	**0.004**
*Conyza* sp.	0.622	0.159	–0.021	0.622	0.794	**0.464**	**0.003**
*D. stramonium*	0.504	0.691	0.191	—	0.721	**0.818**	**0.004**
*D. erucoides*	0.162	0.340	0.054	0.431	0.579	**0.470**	**0.007**
*L. serriola*	–0.233	0.767	0.095	–0.233	0.767	0.235	0.500
*L. amplexicaule*	0.762	0.262	0.238	0.762	—	**0.636**	**0.020**
*M. sativa*	–0.049	0.464	0.492	0.069	–0.131	0.410	0.656
*P. rhoeas*	–0.049	—	—	–0.016	—	0	0.905
*Plantago sp*	0.317	0.399	0.317	0.707	—	**0.667**	**0.0007**
*S. marianum*	–0.132	–0.100	–0.132	–0.132	—	0	0.779
*S. oleraceus*	0.484	0.784	0.084	0.817	0.300	**0.615**	**0.008**
*T. pratense*	0.896	—	–0.079	–0.104	0.896	0.4	0.495
*X. strumarium*	0.670	—	–0.330	–0.330	0.670	0.5	0.786
**Total**	**0.422**	**0.690**	**0.308**	**0.619**	**0.667**	**0.185**	**7.6·10^−15^**
**P**	**9.9·10^−4^**	**1.0·10^−5^**	**6.9·10^−3^**	**1.2·10^−4^**	**1.2·10^−2^**	**7.6·10^−15^**	

1 Value of Association index

2 Probability of rejection of the null hypothesis of independence of infection. When significant, AI and P values are shown in bold face. Raw significance probabilities were corrected by the sequential Bonferroni method for multiple independent tests as indicated in [60].

## Discussion

Most efforts to understand the population biology of pathogens have focussed on specialist pathogens, and population biologists have successfully developed a formal understanding of the dynamics and evolution of single-host pathogens. However, most pathogens of humans, animals and plants are multi-host pathogens [1]–[3], [20]. As stated by Woolhouse et al. [1] “understanding the more complex population biology of multi-host pathogens will be one major challenge in the 21st century “. There is evidence that within an ecosystem the prevalence of multi-host pathogens may differ largely for the different species of their host range [e.g., [21]–[25]]. Similarly, there is evidence of large differences in the prevalence on a host species of the various pathogens that are able to infect it [e.g., [26]–[29]]. However, no attempt has been made, to our knowledge, to analyse if differences in the distribution of multi-host pathogens over their hosts are random or if there are associations between hosts and pathogens. The uncovering of associations between hosts and pathogens would be highly relevant to understand and model the population biology of multi-host pathogens, and for understanding the phenomenon of generalism itself.

We present here indices and tests to analyse if there is association between multi-host pathogens and their hosts. The proposed indices of selectivity for the pathogen and for the host measure the degree of association between hosts and pathogens. The tests analyse the homogeneity of distribution of a pathogen over different host species or populations, and of different pathogens on a host, and analyse how significantly the values of the indices departs from zero (i.e. no association). The literature on pathogen ecology does not abound with data on the prevalence of various pathogens on various hosts. Hence, we have applied these indices to our unpublished data on the prevalence of five insect-borne plant viruses on 21 species of wild plants in an agroecosystem in central Spain over a three year period.

The analysis of the prevalence of the different viruses in each host species by the homogeneity test that we propose, shows that half of the analysed plant species showed an index of selectivity of the host (ISH) significantly different from zero. The distribution of the host species showing virus selectivity was not related to taxonomy, habitat (fallow fields, edges or wastelands), seasonality or vegetative cycle (annual vs. perennial) (not shown). Interestingly, there was a positive correlation between the ISH and the average virus prevalence for these 21 host plant species, showing that the more selective hosts are more prone to be virus-infected, obviously by the virus(es) that better infects them. This phenomenon suggests that in spite that each host encounters a wide array of pathogens, mechanisms of escape and/or resistance [30] to some of them would operate, which could explain their selectivity. In fact, contingency analysis of counts of infected hosts by different viruses, suggest that different viruses specialise on different hosts.

The analysis of the homogeneity of prevalence of a virus over its host species showed that for three of the five analysed viruses there was a significant host association, i.e., the value of the index of selectivity for the pathogen (ISP) significantly departed form zero. One major and unexpected finding of the analysis was that there was a positive and highly significant correlation between the value of the ISP and the prevalence of the viruses. The value of the ISP was not conditioned by the number of host plant species infected by each virus, as there was no correlation (r = 0.60, P = 0.173 in a Spearman rank correlation test) between ISP and the number of plant species that each virus infected in the analysed system i.e., the more selective viruses were not those infecting a smaller number of plant species. Thus, the more host-selective viruses were those that did best in the analysed ecosystem. This result could be highly relevant for understanding the evolution of generalism in pathogens. Although most described pathogens are generalists, the advantages of generalism are poorly understood. A generalist strategy provides the pathogen with more opportunities for transmission and survival, but it is predicted that evolution would favour specialism, because pathogen-host co-evolution could result in functional trade-offs that would limit the generalist fitness in any one host [1], [31]–[34]. Our results are compatible with the hypothesis that specialism is advantageous for pathogens, as host selectivity is the rule for the analysed set of generalist viruses, and the more host selective is the virus, the more successful its strategy. Hence, our results could suggest that for generalist pathogens a degree of host specialisation, i.e. host-selectivity as defined here, is a successful strategy. Host specialisation in generalist pathogens would also be relevant for important issues of host and pathogen biology, as host specialisation will affect host-pathogen co-evolution and co-speciation, would reduce the opportunities for host switches and jumps, thus constraining the evolution of host expansion, and may result in spatial heterogeneity of hosts, thus favouring the stable maintenance of pathogen and host diversity [6], [35]–[37]. In addition, host specialisation may affect the opportunity for different pathogens of sharing a host and, thus, the consequences of multiple infection for pathogen and host evolution, as discussed below.

We propose here also a simple procedure to estimate association among pathogens, which enables to compute an association index whose significance can be tested against the null assumption of independence of infections that follow a binomial distribution. The test was applied to the same data set as above, and the second major contribution of our analysis is the finding that co-infection was mostly non-random and that associations among the five analysed viruses were mostly positive. This result is relevant because co-infection of different pathogens may have important consequences for the pathogens, the infected hosts, and for host-pathogen co-evolution [8], [9], [14]. For viruses, co-infection of a host may result in the generation of new genotypes by recombination or by reassortment of genomic segments between different viral species or strains, often with dramatic changes in host range or pathogenicity. The classical example is the reassortment of avian and human strains of influenza A resulting in novel viruses with pandemic potential [38]–[41], but examples abound for both animal and plant viruses [e.g., [3], [42]–[48]]. In the individual host, co-infection may lead to aggravated disease, often resulting from extracellular cooperativity of independently replicating viruses, by which one virus modulates the host response to infection to the benefit of the other [49], [50]. In addition, direct interactions of different viruses in co-infected cells may result in complementation of highly pathogenic defective genotypes, in increased virus replication or in modified cell and tissue tropisms [e.g., [51]–[57]]. Alternatively, there is also evidence that mixed infections of pathogens result in reduced pathogenicity and less severe disease [17]. Examples from viruses include mixed infection with satellite or with defective interfering nucleic acids [58]. In our data set, association between viruses depended on each particular virus-host system. Hence, data suggest that in some hosts, but not in all, co-infection would be advantageous for some viruses, though the underlying mechanism remains to be analysed.

The analysis here reported of plant virus infection on weeds has uncovered two major features that should be relevant to understand the population biology of viruses: i) the more host-selective viruses do better on the analysed ecosystem, ii) viruses tend to associate positively in co-infected hosts. It would be of high interest to know how general are these features and in which types of pathogens would they occur. The indices and tests that we propose here could be of general use in the analysis of the ecology of pathogens, and we hope that our results would prompt research on the ecology of pathogen-host and pathogen-pathogen associations, as these analyses might uncover pathogen properties relevant to the formal understanding of the population biology of multi-host pathogens.

## Methods

### Indices and tests

We study two factors relative of the distribution of pathogens in different hosts (i.e. different host populations, genotypes, species etc): if there are associations between pathogens and their hosts and if there are associations among pathogens. To analyse these two factors we propose the following tests and indices:

#### Association between pathogens and their hosts

Let us call *N_k_* the number of analysed individuals in host *k* (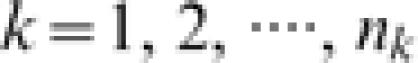) and *X_ik_* the number of these individuals that are infected by pathogen *i* (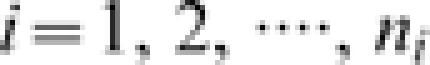). The ***prevalence*** of pathogen *i* in host *k* will be the ratio *P_ik_* = *X_ik_/N_k_*.

The ***average prevalence of pathogen*** *i* over hosts will be
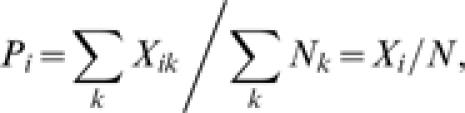
Conversely, the ***average prevalence of the different pathogens*** *in host* *k* can be defined as
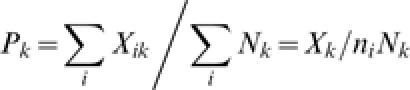
Homogeneity of the prevalence of a pathogen among hosts can be tested by means of a 2*xn_i_* contingency table with elements *X_ik_* and (*N_k_*−*X_ik_*) [59]. Different proportions (i.e. lack of homogeneity) will indicate a property of the pathogen that we will call ***selectivity***. Selectivity will be measured by the Cramer's ***coefficient of contingency*** [59] of the contingency table. If 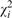 is the chi-squared of the 2*xn_i_* table, the ***index of selectivity of the pathogen*** will be:
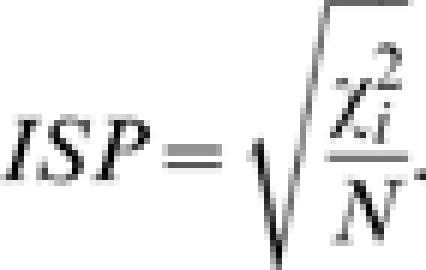
Similarly we can test for homogeneity of the prevalence of the different pathogens in a host, and define the ***index of selectivity of the host*** as:
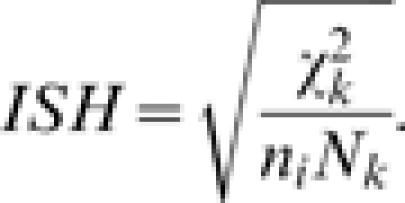
Both of these indices range from zero to one, with zero meaning equal prevalence of the pathogen over hosts, or of pathogens over the same host, i.e. no selectivity for the pathogen or the host.

#### Association between different pathogens

Let us call *Xs_ik_* and *Xa_ik_* the number of analysed individuals of host *k* that are infected only by pathogen *i* (single infections) and by pathogen *i* and at least another one (associated infections), respectively, (*X_ik_* = *Xs_ik_*+*Xa_ik_*).

The frequency of pathogen *i* in host *k* can be estimated as:
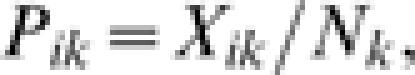
which equals the above defined prevalence. Under the null hypothesis of independence of infection by different pathogens, the probability of a sampled host individual being infected only by pathogen *i* is:
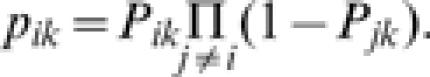
The conditional probability of non-infection by any other pathogen given the presence of pathogen *i* is:
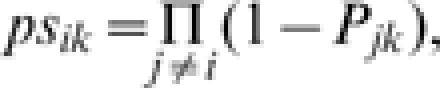
and the conditional probability for the observed multiple infections given the presence of *i* is:
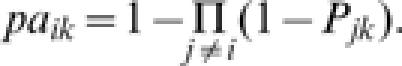
So, under the hypothesis of independence of infection by different pathogens (non-association between pathogens), *Xs_ik_* will be distributed as a binomial with *X_ik_* trials and probability *ps_ik_*.

We define the ***association index*** (*AI*) for pathogen *i* in host *k* as the difference between the proportion of samples that being infected by pathogen *i* are infected also by at least another pathogen (*Xa_ik_/X_ik_*), minus the expectation of this proportion under the null hypothesis (*pa_ik_*). This index has a range from one to minus one and an expected value, under the null hypothesis, of zero. The significance of the observation can be estimated as a one-tail test from the binomial above.

To test for association of different pathogens within a host, or for a given pathogen across different hosts, we follow the same process, as the expectation of a sum of observations will be equal to the sum of their expectations, and the corresponding sums of observations will be binomially distributed given the *X_ik_*.

To single out significant tests in a group, raw significance probabilities were corrected by the sequential Bonferroni method for multiple independent tests as indicated in [60].

### Analyses of virus prevalence in wild plants

Plants were sampled monthly for three years in a horticultural area in central Spain within three habitats characterised by different degrees of human intervention: fallow fields, edges between fields, and wastelands. Plants were sampled systematically along fixed itineraries, with no consideration of symptom expression, as described in Sacristán et al. [21]. Infection by AMV, BWYV, CMV, WMV and TSWV in the sampled plants was analysed by double-antibody sandwich enzyme-linked immunosorbent assay (DAS-ELISA), using commercial antisera (Bio-Rad, Marnes-La-Coquette, France), according to the manufacturer's instructions.

The distribution of the host species showing virus selectivity according to taxonomy, habitat (fallow fields, edges or wastelands), seasonality or vegetative cycle (annual vs. perennial) was analysed by chi-squared tests of 2x N contingency tables, and their significances assessed, as in the rest of tests of this work, by simulation following model III.
